# Successful multidisciplinary treatment including repeated metastasectomy for recurrent squamous cell esophageal carcinoma: a case report

**DOI:** 10.1186/s40792-019-0634-5

**Published:** 2019-05-03

**Authors:** Kosuke Hirose, Hiroshi Saeki, Yuichiro Nakashima, Tomohiro Kamori, Yoshiaki Fujimoto, Tetsuro Kawazoe, Hiroya Matsuoka, Yasuhiro Haruta, Shun Sasaki, Tomoko Jogo, Qingjiang Hu, Yasuo Tsuda, Koji Ando, Eiji Oki, Ryuzo Hiratsuka, Yoshinao Oda, Masaki Mori

**Affiliations:** 1 Department of Surgery and Science, Graduate School of Medical Sciences, Kyushu University, 3-1-1, Maidashi, Higashi-ku, Fukuoka-shi, Fukuoka-ken, 812-8582 Japan; 2 Hiratsuka Gastrointestinal Surgical Clinic, 2-7-5, Jiyugaoka, Munakata-shi, Fukuoka-ken, 811-4163 Japan; 3 Department of Anatomic Pathology and Pathological Sciences, Graduate School of Medical Sciences, Kyushu University, 3-1-1, Maidashi, Higashi-ku, Fukuoka-shi, Fukuoka-ken, 812-8582 Japan

**Keywords:** Esophageal cancer, Recurrence, Long survival, Multidisciplinary treatment

## Abstract

**Background:**

Recurrences after radical esophagectomy are common. The prognosis for recurrent esophageal cancer is generally poor. Recurrences usually occur between 1 and 3 years of surgery, with the duration of median survival after recurrence ranging from 5 to 10 months. The number of sites and involved organs vary among patients. Consequently, a standard therapeutic strategy has not been established, and the role of surgery in the management of recurrence is unclear.

**Case presentation:**

A 67-year-old man presented with dysphagia 6 months previously and was diagnosed with esophageal squamous cell carcinoma (ESCC) in the upper thoracic region (T2M0M0, stage IB), for which he underwent thoracoscopy-assisted esophagectomy and lymphadenectomy. Adjuvant chemotherapy was not prescribed. Three years after the operation, he developed a solitary metastasis in the left lung, requiring segmentectomy followed by chemotherapy with combined cisplatin (CDDP) and 5-fluorouracil (5-FU). The following year, a metastatic lesion was recognized in the right lung, invading the chest wall, for which he underwent partial lobectomy with local chest wall resection. Multiple mediastinal and abdominal lymph node (LN) metastases were detected in the right lung a year later, which necessitated chemoradiation to a dose of 50.4 Gy with concomitant CDDP and 5-FU. Post-treatment computed tomography (CT) showed a good response. Positron emission tomography (PET)-CT revealed a reduction in the metastatic LNs with no fluoro-deoxy-glucose (FDG) uptake. The following year, metastases were detected in the left cervical LNs. Owing to the limited extent of metastases, resection was followed by chemoradiation to a dose of 50 Gy with CDDP and 5-FU. The following year, metastases were detected in the mediastinal LNs; chemotherapy was administered with nedaplatin and docetaxel. The follow-up CT and PET-CT demonstrated complete disappearance of the tumor, and the patient is currently surviving without recurrence for 11 years from the first curative operation.

**Conclusions:**

This case demonstrates that aggressive multidisciplinary treatment including surgery and radiation to achieve local control could be a meaningful treatment strategy in cases with limited and slowly occurring recurrences.

## Background

Esophageal carcinoma is the sixth leading cause of cancer-related deaths in Japan [1]. Advances in surgical techniques and preoperative management have led to improved surgical outcomes. However, patients with advanced disease often experience recurrence, even after curative surgery [2–5]. The recurrence rate after curative surgery ranges from 28 to 47% [6]. The prognosis of the patients with organ recurrences, including the liver, lung, and bone, is particularly poor [7], with the duration of median survival from diagnosis to recurrence ranging between 5 and 10 months [6].

We had previously reported that the recurrence rate at 1 and 2 years after surgery was 71% and 84%, respectively [8]. In terms of the patterns of recurrence, locoregional, hematogenous, and mixed types were seen in 54%, 36%, and 10% of the patients, respectively [8], which reflected the trends in previous reports [9–13]. In previous reports, lymphatic recurrence was most common, followed by hematogenous recurrence. However, hematogenous recurrence was detectable earlier than lymphatic recurrence [14]. Surgery plays little role in the management of hematogenous metastases involving the lung and liver, as systemic metastases often co-exist, and the rate of tumor growth is rapid [15]. In the case of pulmonary metastases, a report suggested that metastasectomy could optimize prognosis [16]. However, the role of metastasectomy in recurrences remains unclear.

Here, we report a rare case surviving for 11 years after successful control of limited and slowly repetitive recurrences of esophageal squamous cell carcinoma. This was achieved with multidisciplinary treatment including repeated metastasectomies and chemoradiation.

## Case presentation

**Fig. 1:**
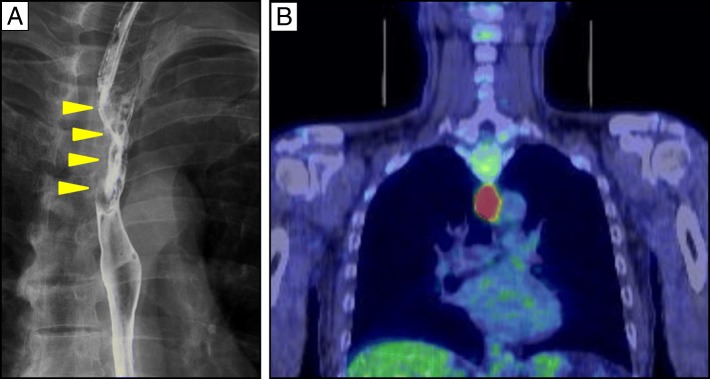
Preoperative images. **a** Esophagography showing the stenosed lesion in the upper thoracic esophagus (arrowheads). **b** PET-CT showing the accumulation of FDG at the lesion

A 67-year-old man was admitted to our hospital with a history of dysphagia for 6 months. Upper gastrointestinal fiber endoscopy revealed a thoracic esophageal lesion. On histopathology, the biopsy specimen of the esophageal lesion revealed squamous cell carcinoma (SCC). Esophagography showed a localized lesion in the upper middle thoracic esophagus (Fig. 1a), and PET-CT showed no distant or local lymph node metastases (Fig. 1b). A preoperative diagnosis of upper thoracic esophageal squamous cell carcinoma (ESCC) of clinical stage T2N0M0 (stage IB) was made based on the TNM classification of the Union for International Cancer Control (UICC) [17]. He then underwent thoracoscopy-assisted esophagectomy and lymph node dissection, with reconstruction using a gastric tube through the retrosternal route. On histopathology, the resected specimen revealed well to moderately differentiated squamous cell carcinoma with invasion of the muscularis propria. According to the Japanese Classification of Esophageal Cancer, the tumor had an infiltrative type b growth pattern, with lymphatic (ly) 2, and venous invasion (v) 1. Intramural metastasis was not seen, and the resected margin was adequate. There were no metastases in the resected LNs, and the tumor was finally staged as T2N0M0 (stage II according to the Japanese Classification of Esophageal Cancer). The postoperative course was uneventful, and he was discharged without any complications. He received no adjuvant therapy in view of pathological stage T2N0M0 (stage IB) disease. He was regularly followed up monthly for 3 months after surgery and at 6 months thereafter. CT scanning was used to check for recurrences, twice a year.

**Fig. 2:**
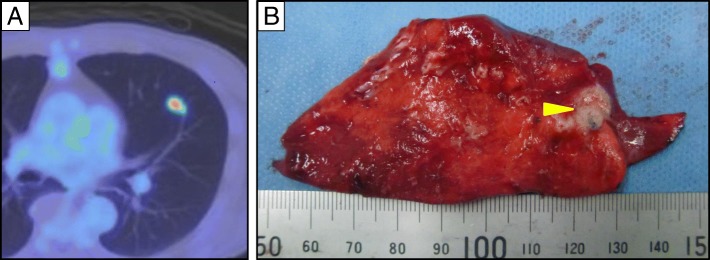
PET-CT and resected specimen of the left lung. **a** PET-CT showing the accumulation of FDG in the metastatic lesion in the left lung. **b** Specimen of the lung after metastasectomy (arrowhead shows the lesion)

At 3 years after surgery, a solitary metastatic lesion was detected by CT and PET-CT in the upper lobe of the left lung (Fig. 2a). A segmentectomy of the left lung was performed (Fig. 2b), followed by chemotherapy with CDDP and 5-FU. Histopathological examination of the resected specimen revealed moderately differentiated squamous cell carcinoma. The well-demarcated nodular lesion consisted of squamoid cancer cells proliferating in nests, with tumor necrosis. The pathologists suggested that the findings were more in favor of metastatic squamous cell carcinoma than primary lung cancer.

**Fig. 3:**
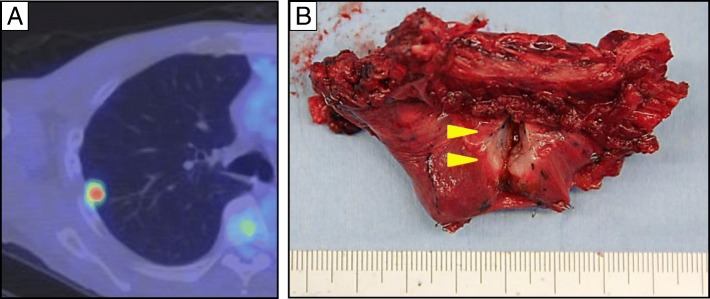
PET-CT and specimen of right lung metastasis invading chest wall. **a** PET-CT showing the metastatic lesion. **b** Resected specimen showing the ashen lesion invading the chest wall (arrowheads)

The year after the lung surgery, a metastatic lesion was detected on CT and PET-CT in the upper lobe of the right lung, invading the chest wall (Fig. 3a). This was managed with a partial lobectomy of the right lobe with local chest wall resection (Fig. 3b). The specimen revealed squamous cell carcinoma and contained lung tissue with the proliferation of severely atypical squamoid cells, arranged in nests. The histologic findings were similar to the previously resected lung specimen, and it was diagnosed as a metastasis from ESCC.

**Fig. 4:**
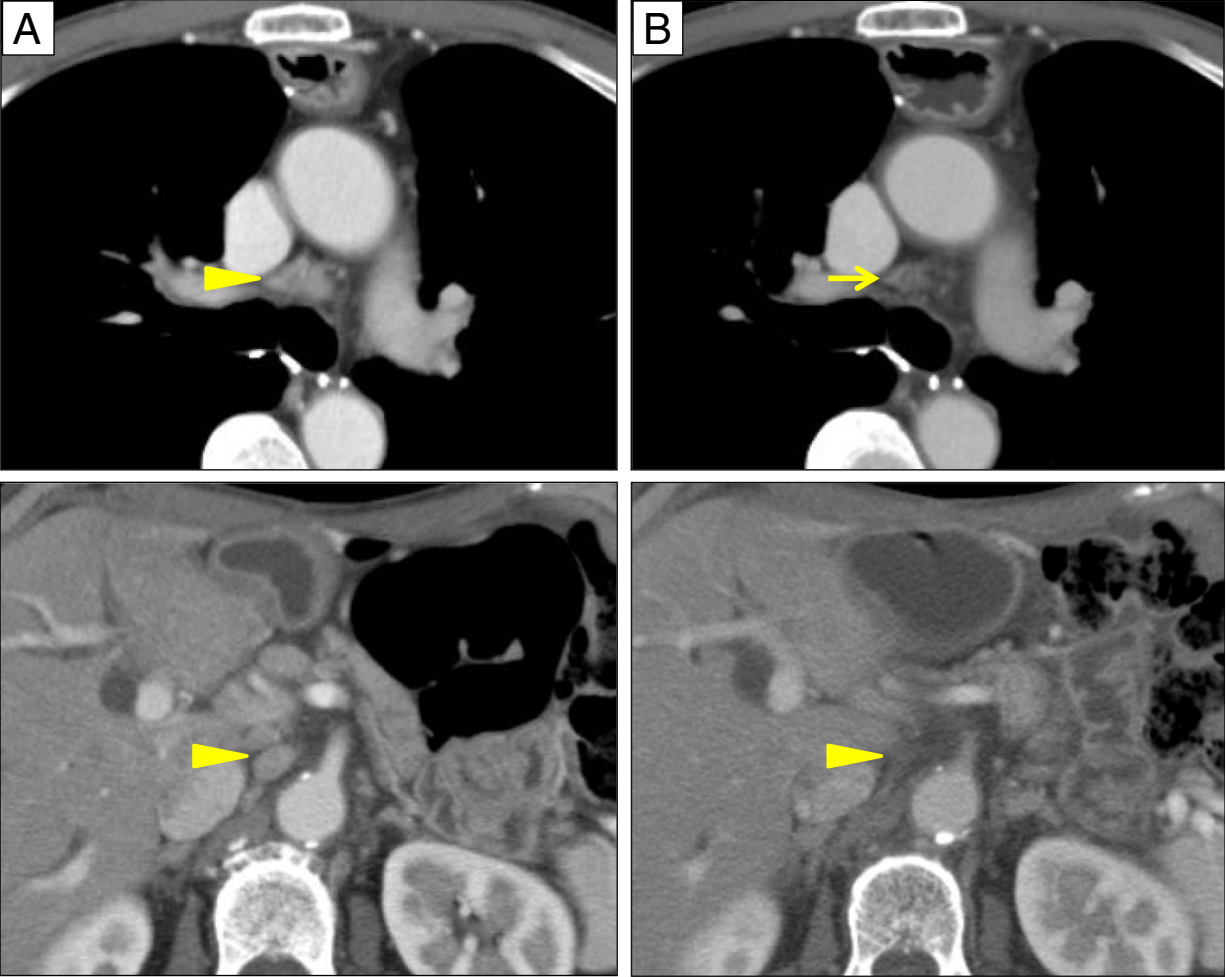
CT images showing metastases in multiple mediastinal and intra-abdominal LN. **a** Before chemoradiation (arrowhead). **b** After treatment, showing mediastinal LNs decreased and intra-abdominal LN disappeared (arrowhead)

The year after right lobectomy, follow-up CT revealed multiple LN metastases in the mediastinum, right supraclavicular region, right axilla, and abdomen (Fig. 4a); PET-CT revealed increased uptake of FDG in the intra-abdominal LN, with a maximum standardized uptake value (SUVmax) of 6.1. The metastatic LNs were managed with chemoradiation to a dose of 50.4 Gy with concurrent CDDP and 5-FU. A partial response was achieved (Fig. 4b).

**Fig. 5:**
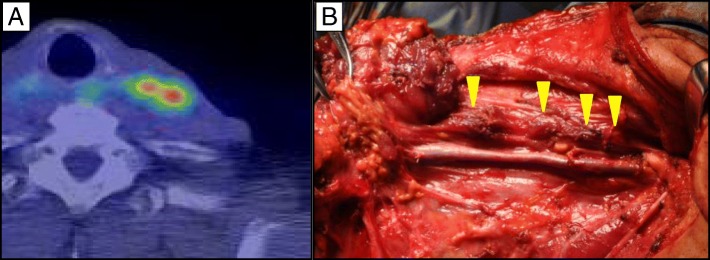
PET-CT showing left cervical locoregional LN metastases. **a** Before resection. **b** Intraoperatively (arrowheads show metastatic LNs)

Locoregional LN metastases in the left cervical region were detected in the year after chemoradiation (Fig. 5a). As the patient had a good performance status, and the nodes were operable at ease, they were resected. (Fig. 5b). Histopathologic examination of the resected LN in the left neck and supraclavicular LNs revealed metastatic cancer cells in 9 of 16 LN. He then received additional chemoradiation to a dose of 50.0 Gy with CDDP and 5-FU.

**Fig. 6:**
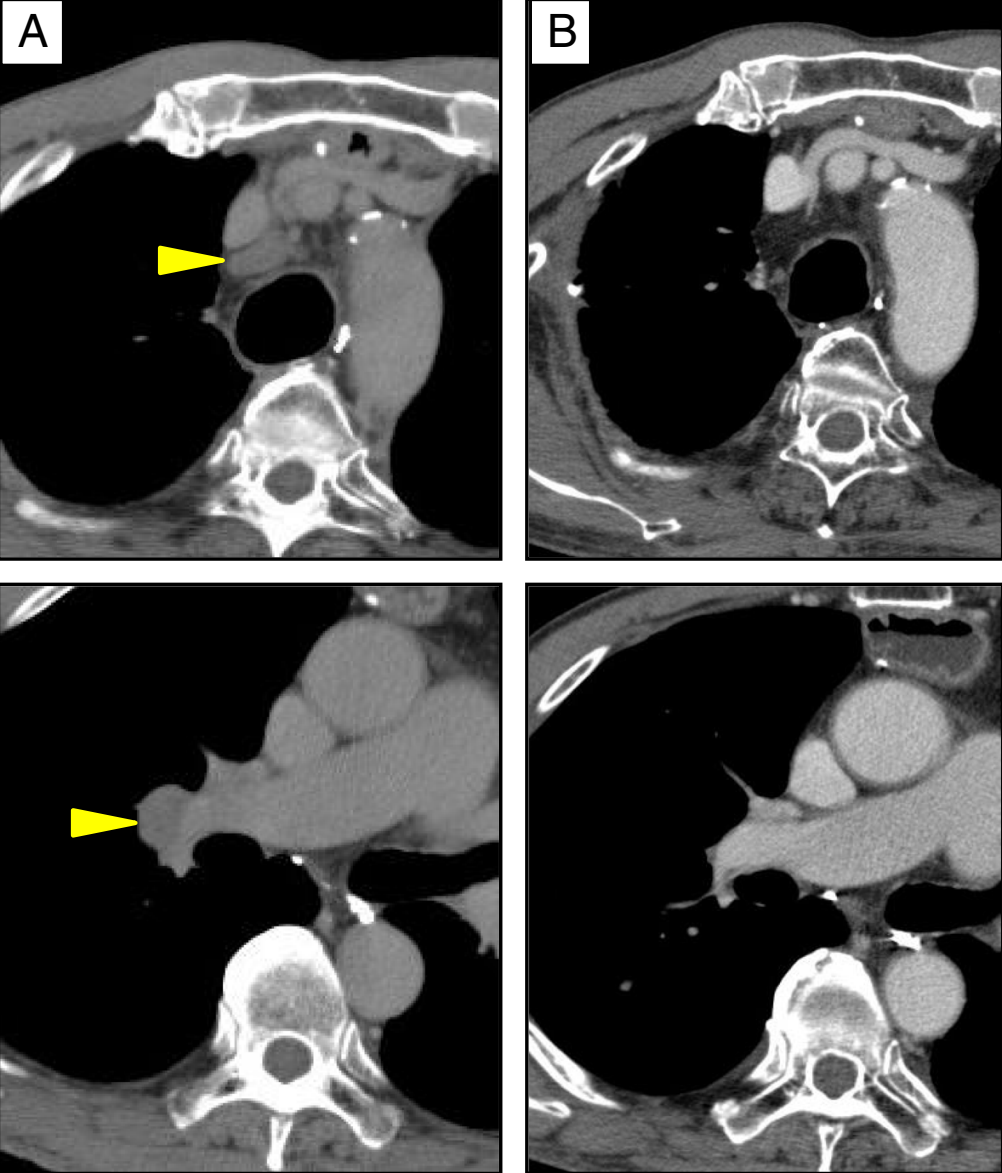
CT images showing LN metastases at mediastinum and hilum. **a** Before chemotherapy (arrowheads). **b** After treatment (the LNs have disappeared)

The fifth recurrence occurred in the LNs of the mediastinum and right hilum, a year after the cervical nodes were treated (Fig. 6a). Chemotherapy was administered with an alternative regimen, combining nedaplatin and docetaxel. The follow-up CT scans after chemotherapy revealed complete response (Fig. 6b). At present, 11 years after esophagectomy, he is still alive with good disease control.

## Discussion

**Fig. 7:**
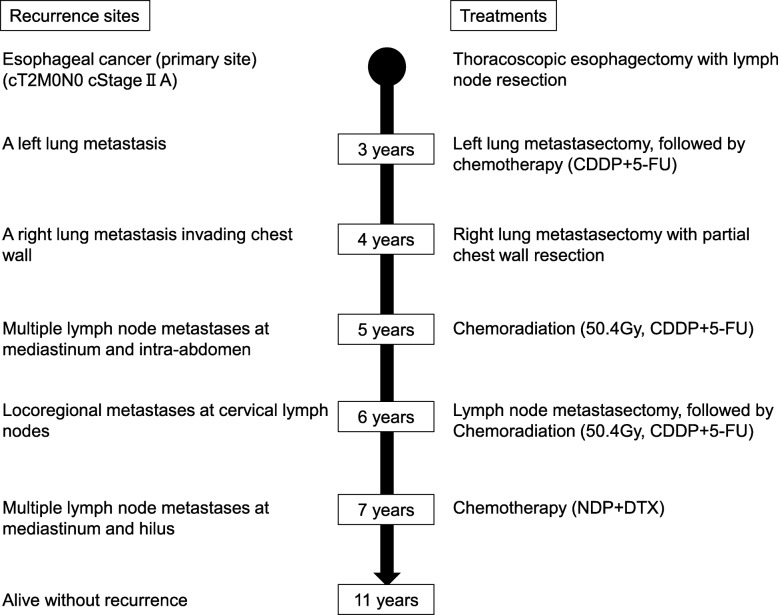
Schematic representation of the clinical course showing recurrent sites and their management

As shown in Fig. 7, this patient experienced locoregional recurrence 5 times in the course of treatment and underwent surgery thrice. Aggressive multidisciplinary treatment including surgery and chemoradiation for multiple recurrences has successfully achieved control of his cancer, and he is still alive.

Here, we reported a case where long-term survival was accomplished despite multiple recurrences of ESCC. Various clinical patterns of recurrence are observed in ESCC. However, only a few trials have studied post-recurrence treatments on a large scale. There are therefore no recommended or standardized guidelines for treatment in these clinical situations [1].

In the present case, the resected specimen of primary ESCC revealed well-to-moderately differentiated squamous cell carcinoma on histopathology. According to the Japanese Classification of esophageal cancer, the tumor had an infiltrative growth pattern of type b, with lymphatic (ly) 2, and venous invasion (v) 1. Tumor diameter (> 4 cm), poor differentiation, and pN3-4 have been reported as prognostic factors in ESCC [18]. Other reports identified postoperative complications of grades 3–4 and adenocarcinoma histology as prognostic factors [19]. Since the present case had no characteristics predictive of a miserable prognosis, the patient was offered intensive treatment for the recurrences. The diagnosis 3metastases is sometimes difficult. However, in this case, the histological features of each resected specimen were similar to the primary ESCC, and they were diagnosed as metastases.

As explained, the first recurrence was observed in the periphery of the left lung at 3 years after the first operation. This progression was relatively slower than that reported in previous reports [8, 9, 20]. The lung is one of the most common organs to which esophageal cancers metastasize [21], but lung metastases often occur concurrently with metastases to other organs or involve multiple metastases at both lungs [9]. Surgical resection for lung metastases is therefore rarely performed [16]. The general outlook of patients after resection of lung metastases is believed to be extremely poor, with reported mean survival periods ranging from 6 to 10 months [22]. However, this case showed that pulmonary metastasectomy might provide favorable outcomes in recurrent cases if the extent of disease is limited and the tumor is non-aggressive.

Unlike in lung metastases, patients with metastases to the locoregional LNs have been reported to achieve longer survival with multidisciplinary treatment, which included resection of the involved nodes [9, 22, 23]. The standard of therapy for recurrent LN metastases has not been defined owing to variations in recurrence patterns, and clear recommendations on the use of chemoradiation or surgery are not available [1]. In our practice, locoregional LN recurrences are resected upfront if operable, followed by chemotherapy to ensure no further recurrences. If surgical resection is not feasible owing to poor performance status, organ dysfunction, or inoperability of metastatic lesions, radiation therapy is administered as appropriate. Nakamura et al. reported that multimodality treatment including local management and chemotherapy could improve outcomes more effectively than chemotherapy alone in cases of recurrent LN metastases after curative esophagectomy [24]. In the present case, the metastatic mediastinal, intra-abdominal, and hilar LN posed difficulties for complete resection, requiring combined chemotherapy and radiation.

Past reports have discussed the efficacy of multidisciplinary treatment for recurrences after curative surgery in esophageal cancer. Iitaka et al. reported on the efficacy of multimodal treatment in a case of ESCC with multiple recurrent lesions [25]. Their patient received combined chemotherapy, radio-frequency ablation, and surgery for the recurrences in the liver and lung after curative esophagectomy. Consequently, the patient survived for 29 months after the primary operation. Suzuki et al. reported on the successful management of recurrent esophageal cancer in a patient with recurrence in the para-aorta LN and liver [26]; the patient received chemoradiotherapy and chemotherapy, both systemically and via hepatic arterial infusions. He survived for about 6 years after the primary esophagectomy with these multidisciplinary treatments. In a case series described by Chen et al., five patients with lung metastases from esophageal carcinoma were treated with pulmonary resection. These patients had metastases from esophageal carcinoma after curative esophagectomy [16]; the median overall survival was 24 months (range 13–90 months). The authors suggested that patients with solitary pulmonary metastases were good candidates for surgery and had a favorable prognosis. The present case demonstrates long-term survival with multidisciplinary treatment for sequential recurrences in the lung and LNs, and supports the efficacy of multimodal and intensive therapy for multiple recurrences in the LNs and lungs.

The patient outcomes of the cases in which intensive or local treatments are particularly effective is a matter of interest. A report by Ghaly et al. described a case where definitive therapy was offered for an isolated recurrence after esophagectomy [27]. They studied 241 patients with recurrence after R0 esophagectomy; 56 patients received definitive treatment for isolated esophageal cancer (EC) recurrences, of whom 31 were treated surgically, and 25 patients received definitive chemoradiotherapy alone. The median survival after recurrence in the former group was 24.5 months (range 12–36 months) and that of the latter group was 28 months (range 12–40 months). Among the 56 patients who had definitive treatments for recurrence, 31 patients (55.4%) survived longer than 2 years after recurrence. In a case series of 42 patients, Kato et al described the treatment of recurrent or residual esophageal SCC with resection, after definitive chemoradiotherapy or surgery [28]. A total of 33, 6, and 3 patients underwent resections of LNs, lung, and other recurrent tumor sites, respectively. Among them, 9 survived more than 3 years, of whom 4 had undergone salvage abdominal lymphadenectomy, 3 underwent resection for solitary lung recurrence, and 2 had undergone surgery at other sites. The authors concluded that surgery for abdominal LN recurrences, LN recurrences outside the radiation field, and solitary lung recurrence was effective. Considering these reports, the numbers of cases with good outcomes to intensive treatment may vary according to the organs involved with recurrence, and the treatment modality offered. LN and lung recurrences are easy to treat intensively; these patients may achieve longer survival.

The interval between each recurrence plays an important role in deciding the therapeutic strategy. To date, only a few studies have investigated the interval between each recurrence, as long-term survival after repeated surgeries or other forms of definitive therapy in cases of recurrent esophageal cancer, is rare. In the present case, the first recurrence was recognized in the third year after primary resection. However, the interval between each of the treated recurrences was within 1 year. Recurrences occurring within 1 year after surgery are common in clinical practice, but in this case, each recurrent lesion was solitary or locoregional, which allowed intensive multidisciplinary treatment.

In summary, we speculate that intensive and multimodality treatment is likely to be particularly effective if the recurrent lesion is solitary or locoregional, and slowly progressive; in cases selected for resection, surgery is particularly effective if the tumor is easy to approach surgically [27].

## Conclusions

We present a case of long-term survival with multidisciplinary treatment for multiple sequential recurrences in ESCC after curative esophagectomy. Despite the poor prognosis of recurrent esophageal cancer, this case demonstrates that multidisciplinary management, including aggressive local therapies, can be particularly effective in cases with localized recurrences, appearing gradually. Cumulative accounts of similar cases, with detailed analyses, are necessary to establish the optimal treatment strategy for recurrences in ESCC.

## Abbreviations

5-FU: 5-Fluorouracil
CDDP: Cisplatin
CT: Computed tomography
ESCC: Esophageal squamous cell carcinoma
FDG: Fluoro-deoxy-glucose
LN: Lymph node
PET-CT: Positron emission tomography
SCC: Squamous cell carcinoma

## References

[1] Kuwano H, Nishimura Y, Oyama T, Kato H, Kitagawa Y, Kusano M (2015). Guidelines for diagnosis and treatment of carcinoma of the esophagus April 2012 edited by the Japan Esophageal Society. Esophagus.

[2] Saeki H, Morita M, Tsuda Y, Hidaka G, Kasagi Y, Kawano H (2013). Multimodal treatment strategy for clinical T3 thoracic esophageal cancer. Ann Surg Oncol..

[3] Tachibana M, Kinugasa S, Yoshimura H, Shibakita M, Tonomoto Y, Dhar DK (2005). Clinical outcomes of extended esophagectomy with three-field lymph node dissection for esophageal squamous cell carcinoma. Am J Surg..

[4] Saeki H, Tsutsumi S, Tajiri H, Yukaya T, Tsutsumi R, Nishimura S (2017). Prognostic significance of postoperative complications after curative resection for patients with esophageal squamous cell carcinoma. Ann Surg..

[5] Saeki H, Nakashima Y, Zaitsu Y, Tsuda Y, Kasagi Y, Ando K (2016). Current status of and perspectives regarding neoadjuvant chemoradiotherapy for locally advanced esophageal squamous cell carcinoma. Surg Today..

[6] Miyata H, Yamasaki M, Kurokawa Y, Takiguchi S, Nakajima K, Fujiwara Y (2011). Survival factors in patients with recurrence after curative resection of esophageal squamous cell carcinomas. Ann Surg Oncol..

[7] Quint LE, Hepburn LM, Francis IR, Whyte RI, Orringer MB (1995). Incidence and distribution of distant metastases from newly diagnosed esophageal carcinoma. Cancer..

[8] Sugiyama M, Morita M, Yoshida R, Ando K, Egashira A, Takefumi O (2012). Patterns and time of recurrence after complete resection of esophageal cancer. Surg Today..

[9] Nakagawa S, Kanda T, Kosugi S, Ohashi M, Suzuki T, Hatakeyama K (2004). Recurrence pattern of squamous cell carcinoma of the thoracic esophagus after extended radical esophagectomy with three-field lymphadenectomy. J Am Coll Surg..

[10] Natsugoe S, Okumura H, Matsumoto M, Uchikado Y, Setoyama T, Uenosono Y (2006). The role of salvage surgery for recurrence of esophageal squamous cell cancer. Eur J Surg Oncol..

[11] Kato H, Nakajima M, Sohda M, Tanaka N, Inose T, Miyazaki T (2009). The clinical application of 18F-fluorodeoxyglucose positron emission tomography to predict survival in patients with operable esophageal cancer. Cancer..

[12] Morita M, Yoshida R, Ikeda K, Egashira A, Oki E, Sadanaga N (2008). Advances in esophageal cancer surgery in Japan: an analysis of 1000 consecutive patients treated at a single institute. Surgery..

[13] Kyriazanos ID, Tachibana M, Shibakita M, Yoshimura H, Kinugasa S, Dhar DK (2003). Pattern of recurrence after extended esophagectomy for squamous cell carcinoma of the esophagus. Hepatogastroenterology..

[14] Morita M, Kuwano H, Ohno S, Furusawa M, Sugimachi K (1994). Characteristics and sequence of recurrent patterns after curative esophagectomy for squamous cell carcinoma. Surgery..

[15] Carlisle JG, Quint LE, Francis IR, Orringer MB, Smick JF, Gross BH (1993). Recurrent esophageal carcinoma: CT evaluation after esophagectomy. Radiology..

[16] Chen F, Sato K, Sakai H, Miyahara R, Bando T, Okubo K (2008). Pulmonary resection for metastasis from esophageal carcinoma. Interact Cardiovasc Thorac Surg..

[17] Sobin LH, Gospodarowicz MK, Wittekind C, editors. TNM classification of Malignant Tumors (7th edition). UICC International Union Against Cancer: Wiley-Blackwell; 2009.

[18] Hsu P, Chen H, Huang C, Liu C, Hsieh C, Hsu H (2017). Patterns of recurrence after oesophagectomy and postoperative chemoradiotherapy versus surgery alone for oesophageal squamous cell carcinoma. Br J Surg..

[19] Luc G, Gronnier C, Lebreton G, Brigand C (2015). Predictive Factors of Recurrence in Patients with Pathological Complete Response After Esophagectomy Following Neoadjuvant Chemoradiotherapy for Esophageal Cancer : A Multicenter Study. Ann Surg Oncol..

[20] Dresner SM, Griffin SM (2000). Pattern of recurrence following radical oesophagectomy with two-field lymphadenectomy. Br J Surg..

[21] Enzinger PC, Ilson DH, Kelsen DP (1999). Chemotherapy in esophageal cancer. Semin Oncol..

[22] Bhansali MS, Fujita H, Kakegawa T, Yamana H, Ono T, Hikita S (1997). Pattern of recurrence after extended radical esophagectomy with three-field lymph node dissection for squamous cell carcinoma in the thoracic esophagus. World J Surg..

[23] Jingu K, Nemoto K, Matsushita H, Takahashi C, Ogawa Y, Sugawara T (2006). Results of radiation therapy combined with nedaplatin (cis-diammine-glycoplatinum) and 5-fluorouracil for postoperative locoregional recurrent esophageal cancer. BMC Cancer..

[24] Nakamura T, Ota M, Narumiya K, Sato T, Ohki T, Yamamoto M (2008). Multimodal treatment for lymph node recurrence of esophageal carcinoma after curative resection. Ann Surg Oncol..

[25] Iitaka D, Shiozaki A, Fujiwara H, Ichikawa D, Okamoto K (2013). A case involving long-term survival after esophageal cancer with liver and lung metastases treated by multidisciplinary therapy : report of a case. Surg Today..

[26] Suzuki T, Izumi Y, Miura A, Kato T, Kawada K (2008). Successful management of the recurrent esophageal cancer following esophagectomy at a different time with combined local treatment of chemotherapy and hepatic arterial infusion chemotherapy. Jpn J Cancer Chemother..

[27] Ghaly G, Harrison S, Kamel MK, Rahouma M, Nasar A, Port JL (2018). Predictors of Survival After Treatment of Oligometastases After Esophagectomy. Ann Thorac Surg..

[28] Kato F, Monma S, Koyanagi K, Kanamori J, Daiko H, Igaki H, et al. Long-term outcome after resection for recurrent oesophageal cancer. 2018;10:2691–9. 10.21037/jtd.2018.05.17 PMC6006052 29997931

